# Transplant Trial Watch

**DOI:** 10.3389/ti.2024.13746

**Published:** 2024-09-30

**Authors:** John M. O’Callaghan, Simon Knight, Keno Mentor

**Affiliations:** ^1^ Department of Transplant Surgery, University Hospitals Coventry & Warwickshire, Coventry, United Kingdom; ^2^ Centre for Evidence in Transplantation, Nuffield Department of Surgical Sciences, University of Oxford, Oxford, United Kingdom; ^3^ Oxford Transplant Centre, Churchill Hospital, Oxford, United Kingdom

**Keywords:** randomised controlled trial, kidney transplantation (KT), hypothermia, normothermia, Treg

To keep the transplantation community informed about recently published level 1 evidence in organ transplantation ESOT and the Centre for Evidence in Transplantation have developed the Transplant Trial Watch. The Transplant Trial Watch is a monthly overview of 10 new randomised controlled trials (RCTs) and systematic reviews. This page of Transplant International offers commentaries on methodological issues and clinical implications on two articles of particular interest from the CET Transplant Trial Watch monthly selection. For all high quality evidence in solid organ transplantation, visit the Transplant Library: www.transplantlibrary.com.

**Randomised Controlled Trial 1**

Fixed Hypothermia for Expanded Criteria Organ Donors in Kidney Transplantation in France (HYPOREME): A Multicentre, Randomised Controlled Trial.

*by The HYPOREME Trial Group. The Lancet Respiratory Medicine 2024 [record in progress].*

## Aims

This study aimed to examine the effect of donor hypothermia versus normothermia on the risk of delayed graft function.

## Interventions

Donors were randomised to either hypothermia or normothermia.

## Participants

365 expanded criteria kidney donors with death diagnosed based on neurological criteria and 526 graft recipients.

## Outcomes

The primary endpoint was proportion of renal transplant patients with delayed graft function. Secondary endpoints in donors were the number of organs recovered and transplanted, blood pressure, body temperature, kidney function, need for vasopressors and inotropes, total volume of fluids administered, and adverse events. Secondary endpoints for recipients were graft function, length of hospital stay, vital status and adverse events.

## Follow-Up

1 year after transplantation.

## CET Conclusion

*by Simon Knight*

This multicentre, randomised trial recruited 365 extended-criteria DBD donors in 53 intensive care units in France. Donors were randomised to hypothermia (34°C–35°C) or normothermia. Hypothermic machine preservation was routinely used for kidneys from recruited donors. The focus of the study was the outcomes of the kidney transplant recipients. Primary endpoint was incidence of delayed graft function, which was not significantly different between groups. A small, statistically significant difference was seen in 1-year graft function, with lower serum creatinine and higher creatinine clearance. One thing that is not clear from the manuscript is how many of the donors donated organs other than kidneys, and what the outcomes were for these organs. Clearly, any donor intervention has the potential to impact all donated organs, and so this should be mandatory outcome reporting for donor intervention studies. Otherwise, the study is well designed and reported, with block randomisation, allocation concealment and blinding of outcome assessment. The clinical significance of the differences in kidney function at 1 year is small but given the simplicity of the intervention and evidence for safety, it adds to the existing evidence that donor hypothermia may be beneficial to organ recipients.

## Jadad Score

3.

## Data Analysis

Modified intention-to-treat analysis.

## Allocation Concealment

Yes.

## Trial Registration

ClinicalTrials.gov - NCT03098706.

## Funding Source

Non-industry funded.

**Randomised Controlled Trial 2**

Effect Late Treatment with Autologous Expanded Regulatory T-Cell Therapy After Alemtuzumab Induction Is Safe and Facilitates Immunosuppression Minimization in Living Donor Renal Transplantation.

*by Brook, M. O., et al. Transplantation 2024 [record in progress].*

## Aims

The aim of this study was to examine the effect of delayed infusion of autologous expanded regulatory T-cell (Treg) following alemtuzumab induction in living donor kidney transplant recipients.

## Interventions

Participants were randomised to either the Treg therapy arm or to standard immunosuppression alone.

## Participants

7 living donor kidney transplant patients.

## Outcomes

The primary endpoints of interest were patient survival, graft survival and the incidence of biopsy-confirmed acute rejection events.

## Follow-Up

18 months post-transplantation.

## CET Conclusion

*by Keno Mentor*

The Transplantation Without Overimmunosuppression (TWO) study was originally designed to investigate the efficacy of regulatory T cells (Treg) therapy to enable the reduction of long-term immunosuppression. Treg cells were to be infused 6 months after kidney transplantation with Alemtuzumab induction therapy. However, during the COVID-19 pandemic, Alemtuzumab use was suspended because of safety concerns and the trial was re-designed based on Basiliximab induction therapy. Prior to this change, 7 patients received therapy as per the original protocol – these results are presented in this study as a proof-of-concept analysis. 3 patients were randomised to the Treg therapy group with elimination of MMF and reduction of tacrolimus, and 4 to the standard immunosuppression (alemtuzumab induction, Tacrolimus & MMF therapy) group. The patients were followed up for 18 months. MMF elimination was achieved in all patients in the treatment group, but although tacrolimus doses were decreased, average trough levels were equal between the two groups. There was 100% graft survival in both groups and no episodes of rejection in the Treg group versus one episode in the standard group. Adverse events were generally low in both groups. With such a small cohort, these results cannot be generalisable, but the study does contribute to and support existing data that demonstrates that Treg therapy is safe and potentially enables reduction of immunosuppression. Larger studies with long-term follow-up are needed to better determine the degree of benefit.

## Jadad Score

3.

## Data Analysis

Strict intention-to-treat analysis.

## Allocation Concealment

Yes.

## Trial Registration

International Standard Randomised Controlled Trial Number registry - 11038572.

## Funding Source

Non-industry funded.

## Clinical Impact Summary

*by John O’Callaghan*

This is a thought-provoking clinical study that set out to use regulatory T-cells after renal transplantation, with alemtuzumab induction and overall reduction in immune suppression over time. The study arm received an infusion of regulatory T-cells at week 26, following which the patients would stop MMF and be maintained on tacrolimus monotherapy. In the control arm all patients would continue tacrolimus and MMF from the time of transplant. All were living donor transplants and low risk immunologically. The target inclusion was 68 patients.

The study commenced prior to the COVID pandemic, during which the use of alemtuzumab was suspended in the United Kingdom, and the study protocol had to be changed. The published study report therefore only includes data from a cohort of 7 patients, 3 of whom were in the cell therapy arm. The regimen in the study arm is interesting, with a protocol biopsy at 26 weeks, after which MMF was stopped. A second protocol biopsy was taken at 38 weeks, and if satisfactory the dose of tacrolimus was taken down to maintain reduced trough levels. An interesting feature of the regimen is the delayed infusion of regulatory T-cells at 6 months after transplant, which targets the lymphocyte repopulation phase.

Unfortunately, the direct clinical impact of the study is significantly reduced by the very much reduced patient inclusion. The 3 patients who received the regulatory T-cell infusion had no haemodynamic or inflammatory reaction. Transplant Survival at 18 months was 100%, with no acute rejection in the study arm, and one patient in the control arm with early acute rejection treated with steroids. One control patient experienced a decline in renal function due to CNI toxicity.

This paper reports on the safety of delayed infusion of regulatory T-cells, at 26 weeks after renal transplantation, and following immune induction with a leukodepleting agent. This will be a proof of concept for future studies in this field.

